# Arterial Stiffening Moderates the Relationship Between Type-2 Diabetes Mellitus and White Matter Hyperintensity Burden in Older Adults With Mild Cognitive Impairment

**DOI:** 10.3389/fnagi.2021.716638

**Published:** 2021-10-25

**Authors:** Madeleine L. Werhane, Kelsey R. Thomas, Katherine J. Bangen, Alexandra J. Weigand, Emily C. Edmonds, Daniel A. Nation, Erin E. Sundermann, Mark W. Bondi, Lisa Delano-Wood

**Affiliations:** ^1^Department of Psychiatry and Behavioral Sciences, University of Washington, Seattle, WA, United States; ^2^VA San Diego Healthcare System, San Diego, CA, United States; ^3^Department of Psychiatry, University of California, San Diego, La Jolla, CA, United States; ^4^SDSU/UC San Diego Joint Doctoral Program in Clinical Psychology, San Diego State University/University of California, San Diego, San Diego, CA, United States; ^5^Department of Psychological Sciences, University of California, Irvine, Irvine, CA, United States

**Keywords:** mild cognitive impairment, white matter hyperintensity volume, blood pressure, arterial stiffness, diabetes

## Abstract

**Background:** Cerebrovascular dysfunction has been proposed as a possible mechanism underlying cognitive impairment in the context of type 2 diabetes mellitus (DM). Although magnetic resonance imaging (MRI) evidence of cerebrovascular disease, such as white matter hyperintensities (WMH), is often observed in DM, the vascular dynamics underlying this pathology remain unclear. Thus, we assessed the independent and combined effects of DM status and different vascular hemodynamic measures (i.e., systolic, diastolic, and mean arterial blood pressure and pulse pressure index [PPi]) on WMH burden in cognitively unimpaired (CU) older adults and those with mild cognitive impairment (MCI).

**Methods:** 559 older adults (mean age: 72.4 years) from the Alzheimer’s Disease Neuroimaging Initiative were categorized into those with diabetes (DM+; CU = 43, MCI = 34) or without diabetes (DM-; CU = 279; MCI = 203). Participants underwent BP assessment, from which all vascular hemodynamic measures were derived. T2-FLAIR MRI was used to quantify WMH burden. Hierarchical linear regression, adjusting for age, sex, BMI, intracranial volume, CSF amyloid, and APOE ε4 status, examined the independent and interactive effects of DM status and each vascular hemodynamic measure on total WMH burden.

**Results:** The presence of DM (*p* = 0.046), but not PPi values (*p* = 0.299), was independently associated with greater WMH burden overall after adjusting for covariates. Analyses stratified by cognitive status revealed a significant DM status x PPi interaction within the MCI group (*p* = 0.001) such that higher PPi values predicted greater WMH burden in the DM + but not DM- group. No significant interactions were observed in the CU group (all *p*s > 0.05).

**Discussion:** Results indicate that higher PPi values are positively associated with WMH burden in diabetic older adults with MCI, but not their non-diabetic or CU counterparts. Our findings suggest that arterial stiffening and reduced vascular compliance may have a role in development of cerebrovascular pathology within the context of DM in individuals at risk for future cognitive decline. Given the specificity of these findings to MCI, future exploration of the sensitivity of earlier brain markers of vascular insufficiency (i.e., prior to macrostructural white matter changes) to the effects of DM and arterial stiffness/reduced vascular compliance in CU individuals is warranted.

## Introduction

Type 2 diabetes (hereby referred to as “diabetes” or DM) has been repeatedly linked to an increased risk for developing dementia in late life (Arvanitakis et al., 2004; Crane et al., 2013). Given that both diabetes and dementia represent chronic, debilitating conditions that are extremely common in our rapidly aging population (Alzheimer’s Association [AA], 2019; Centers for Disease Control and Prevention [CDCP], 2020), there has been a critical push for research aimed at disentangling the nature of their association in order to aid in prognosis and identify potential treatment targets. Cerebrovascular dysfunction remains a putative mechanism by which poor cognitive outcomes occur in diabetes. Dementia in the context of diabetes has been linked to increased cerebrovascular pathology at autopsy, which stands in contrast to dementia without diabetes that on average has greater evidence of other neuropathological changes (e.g., amyloid beta and tau accumulation; Sonnen et al., 2009). Well-aligned with such findings is the observation that the most common neuroradiological finding associated with diabetes is the presence of increased white matter hyperintensity (WMH) burden in the brain (Van Harten et al., 2006). Thought to reflect a highly prevalent form of cerebrovascular disease (i.e., small vessel ischemic damage to deep white matter regions; DeCarli et al., 2005), the presence of increased WMH burden on imaging is associated with greater risk for age-related cognitive decline, mild cognitive impairment (MCI), and dementia (Delano-Wood et al., 2008, 2009; Debette and Markus, 2010; Nation et al., 2013; Bangen et al., 2018).

Structural neuroimaging studies have also demonstrated *in vivo* evidence of cerebral atrophy and accumulation of neurodegenerative pathology in individuals with diabetes, which in turn predict poorer cognitive performance across domains including memory, executive functioning, and processing speed (Tiehuis et al., 2009; Hayashi et al., 2011; Moran et al., 2013). Critically, these white and gray matter alterations observed in individuals with diabetes likely represent end-stage pathological changes to brain parenchyma attributable to chronic cerebrovascular dysfunction, as suggested by prior work reporting links between cognitive functioning and alterations in regional cerebral blood flow (an indicator of cerebral perfusion), but not cortical thickness or regional brain volume, in older adults without dementia (Bangen et al., 2018). Combined, this research highlights the need to identify sensitive, early markers of cerebrovascular dysfunction prior to the development of irreversible brain pathology and cognitive impairment in individuals with diabetes.

While hypertension, specifically, has shown the most consistent association with increased WMH burden (de Leeuw et al., 2002; de Havenon et al., 2019), there is accumulating evidence for arterial stiffness—the decreased elasticity of the arterial wall that occurs in the context of aging—as a sensitive predictor of risk for cerebrovascular disease and dementia. Particularly notable are longitudinal findings that show that increased arterial stiffness at baseline in cognitively unimpaired older adults predicts future accumulation of concomitant white matter disease and neurodegenerative pathology (Ohmine et al., 2008; Mitchell et al., 2011), as well as increased risk for cognitive impairment, functional decline, and dementia diagnosis (Nation et al., 2013, 2016; Werhane et al., 2018). Importantly, arterial stiffness represents one of the earliest indicators of change to vascular wall structure and function in the progression of cardiovascular and cerebrovascular disease (Cohn et al., 2005), and is accelerated in health conditions such as diabetes that negatively impact the integrity of the vascular system (Xu et al., 2016; Chirinos et al., 2019).

Taken together, this literature highlights the potential role of vascular-induced white matter pathology in the development of poor cognitive outcomes in aging individuals with diabetes. Indeed, vascular disease, alterations to white matter integrity, and cognitive impairment are all highly prevalent in older adults, regardless of diabetes status. However, few studies to date have characterized differential relationships between vascular and white matter pathologies, especially in relation to the presence of cognitive impairment, in older adults with and without diabetes. Therefore, we explored relationships between diabetes status (i.e., diabetes absent vs. present) and different vascular hemodynamic measures (i.e., diastolic and systolic BP [BP], pulse pressure index [PPi], and mean arterial BP [MABP]) to the presence of MRI evidence of small vessel cerebrovascular disease (i.e., WMH) in a large sample of well-characterized older adults without dementia. Of particular interest was PPi as an indicator of systemic vascular pathology, given literature to suggest its utility as an easily obtainable blood-pressure based measure of arterial stiffness and reduced vascular compliance (Peng-Lin and Yue-Chun, 2009). In order to understand how relationships between different vascular hemodynamic measures, diabetes status, and WMH burden may vary across the spectrum of cognitive aging to early stages of cognitive impairment, these variables were examined in both cognitively unimpaired older adults as well as those with a diagnosis of MCI.

We hypothesized the following: (1) greater arterial stiffness, as indicated by greater PPi values, would predict greater WMH volume on neuroimaging across cognitive groups, and (2) any observed relationship would be more pronounced in individuals with MCI versus cognitively unimpaired. Additionally, we also predicted that (3) older adults with both diabetes and greater arterial stiffening would demonstrate greater white matter pathology burden compared to those with one or no risk factors (i.e., no diabetes and/or reduced arterial stiffening); and (4) this interactive effect will be observed to a lesser extent in older adults with no evidence of cognitive impairment (i.e., cognitively unimpaired [CU] older adults). These hypotheses were evaluated using data from the Alzheimer’s Disease Neuroimaging Initiative (ADNI).

## Materials and Methods

### The ADNI Dataset

Data used for the present study were obtained from the ADNI database (adni.loni.usc.edu). The ADNI was launched in 2003 by the National Institute on Aging (NIA), the National Institute of Biomedical Imaging and Bioengineering (NIBIB), the Food and Drug Administration (FDA), private pharmaceutical companies and non-profit organizations. The main goal of ADNI has been to determine whether MRI, PET, other biological markers, and clinical and neuropsychological assessment can be combined to measure the progression of MCI and early AD. The Principal Investigator of ADNI is Michael W. Weiner, MD, VA Medical Center and University of California – San Francisco. ADNI is the result of efforts of many co-investigators from a broad range of academic institutions and private corporations, and participants have been recruited from over 50 sites across the United States and Canada. ADNI has been followed by ADNI-GO and ADNI-2. Subjects originally recruited for ADNI-1 and ADNI-GO had the option to be followed in ADNI-2. Data used in the present study were acquired in ADNI-2. For up-to-date information, see www.adni-info.org.

### Participants

The sample comprised 559 dementia-free older adults with or without MCI from ADNI. All participants were between the ages of 55 and 90 years old, had completed at least six years of education, were fluent in Spanish or English, and were free of any significant neurological or psychiatric disease. Information about each participant’s medical history and medications was collected at baseline. All participants underwent neuropsychological testing and brachial BP assessment at baseline. Full criteria for ADNI eligibility and diagnostic classifications are described in detail at http://www.adni-info.org/Scientists/ADNIGrant/ProtocolSummary.aspx.

Participant selection was as follows: Of the 2,952 ADNI participants who completed neuropsychological assessment, 2,393 participants were identified as having no dementia at baseline based on ADNI’s criteria for dementia. Consistent with previous work, we excluded individuals with functional dependence (based on a Functional Assessment Questionnaire [FAQ] score > 5) to ensure that that the final study sample consisted of older adults who could independently complete their activities of daily living (e.g., Thomas et al., 2017; Werhane et al., 2018). Of this sample, 559 participants had available WMH volume data. Within this subsample, the comprehensive neuropsychological criteria for MCI (Jak et al., 2009; Bondi et al., 2014) were used to identify cognitive status, yielding a final analytic sample of 322 CU older adults and 237 with MCI. Participants were then classified as either diabetic (DM+; CU = 43, MCI = 34) or non-diabetic (DM-; CU = 279; MCI = 203) based on their self-reported diabetes diagnosis, presence of glucose-lowering agents in their medical history, and/or fasting blood glucose above American Diabetes Association cutoff values for a diagnosis of diabetes (i.e., > 125 mg/dL). Consistent with our previous work in ADNI (Thomas et al., 2020), the following search terms were used to identify participants with DM at their initial study visit from medical history: diabetes, diabetic, insulin, insulin-dependent diabetes mellitus, and non-insulin dependent diabetes mellitus.

### White Matter Imaging

All participants underwent baseline MR imaging from which total WMH volume was derived. A detailed description of ADNI MR imaging data acquisition and processing can be found online^1^. Briefly, participants were scanned using a 3T MRI scanner. A T1-weighted sequence was acquired using the following parameters: TR = 2300 ms; TE = 2.98 ms; TI = 900 ms; 170 sagittal slices; within plane FOV = 256 × 240 mm^2^; voxel size = 1.1 × 1.1 × 1.2 mm^3^; flip angle = 9*;* bandwidth = 240 Hz/pix. T2 FLAIR scans were also obtained using an echo-planar imaging sequence with the following parameters: TR = 9000ms; TE = 90ms; TI = 2500ms; 42 slices at a thickness 5 mm. T1-weighted and T2 FLAIR scans were both pre-processed through a standardized pipeline and co-registered using cross-correlation. Brain and non-brain tissues were separated via skull-stripping. WMHs were detected using the previously validated, semi-automated Bayesian Markov-Random Field (MRF) method (DeCarli et al., 1999). The skull-stripped T1-weighted image was then non-linearly aligned to a minimum deformation template (MDT), to which the T1, T2 FLAIR, and map of ground-truth FLAIR-based WMH pixels were then warped using the non-linear alignment.

### Blood Pressure Assessment

Seated brachial artery systolic and diastolic BPs were obtained from ADNI participants at their initial study visit. PPi values were calculated for each participant by dividing pulse pressure values (systolic BP – diastolic BP) by systolic BP. PPi allows for the evaluation of the effects of pulse pressure, a common proxy for arterial stiffening, while also removing the effects of systolic pressure. This measure was derived to help disambiguate the potential effects of arterial stiffening versus hypertension in study findings, and allows for the improved evaluation of arterial stiffness and vascular compliance in the context of pathological vascular aging (Peng-Lin and Yue-Chun, 2009). Mean arterial pressure was calculated as diastolic pressure plus one-third the pulse pressure. All arterial BP measurements were taken using a calibrated mercury sphygmomanometer and BP cuff. BP readings were taken from the dominant arm while the participant was in a seated position, with their forearm held horizontally at the level of the fourth intercostal space at the sternum (i.e., the level of the heart).

### Statistical Analyses

Demographic and clinical characteristics by diabetes and cognitive status were examined using linear regression and chi-square tests for continuous and categorical variables, respectively. Hierarchical multiple linear regression was used in order to assess the independent and interactive effects of diabetes status and each vascular hemodynamic measure on total WMH volume within the overall and then within cognitive normal and MCI groups, separately. Parameters of interest in the models included vascular hemodynamic variables (systolic BP, diastolic BP, PPi, and MABP; all continuous and mean centered) and diabetes status (dichotomous; type 2 diabetes absent/present). The outcome of interest was total WMH volumes (continuous), which was log-transformed in order to improve distributional normality. At the first level of each model, age, sex, BMI, amyloid beta 1-42 positivity (Hansson et al., 2018), apolipoprotein E (APOE) ε4 status, and intracranial volume were all entered given well-established associations with vascular aging and WMH volume in the literature. Diabetes status, a vascular hemodynamic variable, and their interaction term were then entered on the second level. Again, separate models were generated for each vascular hemodynamic measure, stratified by cognitive status. Effect sizes were indexed semi-partial r values. The Bonferroni method was used to control for familywise error inflation due to multiple comparisons (statistical significance threshold: *p* < 0.05/8; Bonferroni corrected *p*-value = 0.006). All analyses were performed using R Studio Version 1.1.453 (2009-2018 RStudio, Inc.).

## Results

Participant demographics and clinical characteristics are presented in Table 1. The mean age of the overall sample was 72.4 years (SD: 6.94 years; range: 55.1 to 91.4 years). Mean physiological (i.e., BP [systolic, diastolic], arterial stiffness, MABP) and psychometric scores were in the non-clinical range^2^ both across the entire sample and within diabetes status groups, confirming that the sample was generally healthy with respect to vascular, psychiatric, cognitive, and functional symptomatology. Diabetes status did not significantly differ by cognitive group (CU vs. MCI, *p* = 0.591). The bivariate association between DM status and WMH burden was not significant in the overall sample; however, a significant positive association was observed between diabetes status and WMH burden once covariates were included in an adjusted model (*p* = 0.045). Both bivariate and adjusted associations between PPi and WMH burden were not statistically significant in the sample overall, nor within cognitive subgroups (*p*s > 0.05). Within the CU group, participants with diabetes had fewer years of formal education at the level of a statistical trend (*p* = 0.060) and performed more poorly on a brief screen of global cognitive functioning (Mini Mental Status Exam [MMSE]; *p* = 0.028) relative to non-diabetic participants. Comparatively, within the MCI group, participants with diabetes on average were significantly younger (*p* = 0.005) and had significantly greater BMI values (*p* < 0.001) relative to those without diabetes. No other significant differences by diabetes status were observed within CU or MCI groups across all other demographic characteristics, parameters of interest (PPi, WMH total volume), or variables related to increased vascular and dementia risk (e.g., BP values, depressive symptomology, everyday functioning, genetic risk for dementia, cerebrospinal fluid amyloid beta positivity).

**TABLE 1:** Sample characteristics by diabetes status and cognitive diagnosis.

		DM +	DM-	Differ by DM status?	Differ by cognitive dx?
		
Variable	Cognitive dx	Mean (SD)	Mean (SD)	*p* or χ^2^ value	*p* or χ^2^ value
*N*	MCI	*N* = 34	*N* = 203	–	–
	CU	*N* = 43	*N* = 279	–	–
Age (*years*)	MCI	73.1 (6.89)	69.5 (7.41)	**0.005**	0.358
	CU	72.0 (6.82)	72.2 (6.30)	0.852	–
Education (*years*)	MCI	16.4 (2.54)	15.7 (3.05)	0.182	0.055
	CU	16.5 (2.52)	15.7 (2.50)	0.060	–
Sex (*% male*)	MCI	69.23%	53.55%	0.129	0.168
	CU	58.62%	48.46%	0.297	–
Systolic BP (*mm Hg*)	MCI	137.3 (16.27)	135.1 (19.80)	0.490	0.320
	CU	135.6 (16.96)	134.2 (14.14)	0.781	–
Diastolic BP (*mm Hg*)	MCI	76.0 (9.92)	75.4 (10.63)	0.736	0.383
	CU	75.2 (9.62)	74.9 (8.07)	0.831	–
Pulse Pressure Index (*mm Hg/mm Hg*)	MCI	0.4 (0.07)	0.4 (0.09)	0.600	0.924
	CU	0.4 (0.07)	0.4 (0.08)	0.984	–
Mean Arterial BP (*mm Hg*)	MCI	95.27 (11.50)	96.41 (10.32)	0.560	0.275
	CU	94.9 (98.27)	95.4 (10.26)	0.774	–
BMI (*kg/m^2^*)	MCI	27.0 (4.92)	31.1 (6.36)	< 0.001	0.846
	CU	27.4 (4.77)	28.3 (5.70)	0.245	–
GDS Total Score	MCI	1.6 (1.45)	2.0 (1.62)	0.082	**0.002**
	CU	1.3 (1.40)	1.0 (1.02)	0.289	–
MMSE Score	MCI	27.8 (1.80)	27.8 (1.94)	0.968	** < 0.001**
	CU	28.9 (1.20)	28.4 (2.18)	**0.028**	–
FAQ Score	MCI	3.6 (4.30)	4.0 (4.33)	0.642	** < 0.001**
	CU	0.6 (1.15)	0.58 (1.16)	0.973	–
APOE ε4 status (*%*ε4 +)	MCI	50.00%	51.66%	0.977	** < 0.001**
	CU	44.83%	34.81%	0.092	–
CSF amyloid beta (*% positive)*	MCI	65.38%	65.88%	0.960	** < 0.001**
	CU	37.93%	36.18%	0.851	–
WMH total volume^†^	MCI	0.6 (0.66)	0.6 (0.48)	0.138	** < 0.001**
	CU	0.5 (0.45)	0.4 (0.49)	0.256	–
ICV	MCI	1405.9 (142.10)	1417.0 (147.02)	0.178	0.125
	CU	1393.1 (129.20)	1367.8 (132.50)	0.235	–

*Abbreviations: DM, diabetes mellitus; dx, diagnosis; MCI, mild cognitive impairment; CU, cognitively unimpaired; BP, blood pressure; BMI, body mass index; CDR, clinical dementia rating; GDS, Geriatric Depression Scale; MMSE, Mini Mental State Exam; FAQ, Functional Assessment Questionnaire; APOE, apolipoprotein E; WMH, white matter hyperintensity; ICV, intracranial volume.*

*^†^This variable was log-transformed in order to improve the normality of the distribution. Bold values indicate for *p* ≥ 0.05.*

**FIGURE 1:**
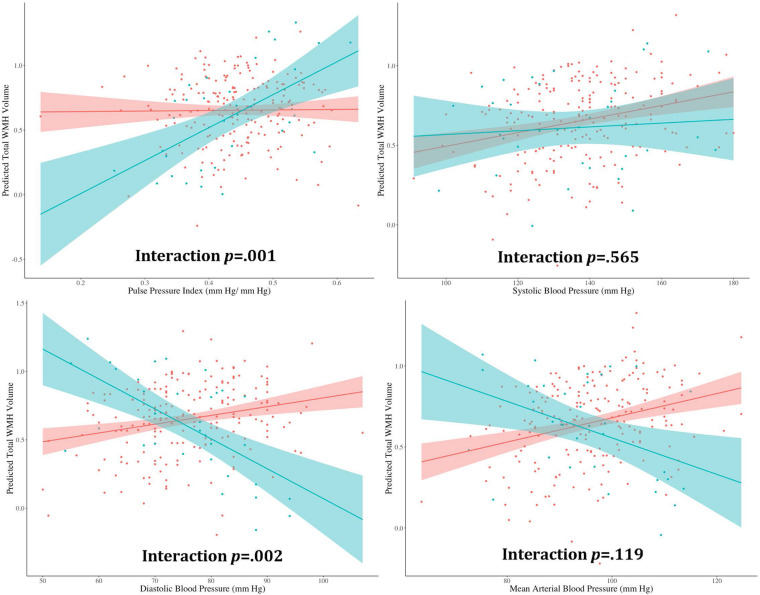
The effect of different vascular hemodynamic measures on the relationship between diabase status and total white matter hyperintensity burden in older adults with mild cognitive impairment. *Note.* DM, diabetes mellitus; WMH, white matter hyperintensity. Within the MCI group, separate hierarchical multiple linear regression models evaluated the interactive effect of diabetes status and each vascular hemodynamic measure on total WMH volumes. All models were adjusted for age, sex, body mass index, intracranial volume, cerebrospinal fluid amyloid beta positivity, and apolipoprotein E ε4 status. PPi significantly modified the relationship between diabetes status and total WMH burden (interaction *p* = 0.001), such that PPi values were associated with greater WMH burden in older adults with MCI and diabetes (β = 1.99, S.E. = 0.943, 95% CI: 0.051–3.928; *p* = 0.045, partial *r* = 0.382), but not their non-diabetes counterparts (*p* > 0.05). Diastolic BP also significantly interacted with diabetes status on total WMH volume (interaction *p* = 0.002). Here, greater diastolic BP values were associated with greater WMH volumes in non-diabetic older adults with MCI (β = 0.008, S.E. = 0.003, 95% CI: 0.155, 2.438; *p* = 0.016, partial *r* = 0.382). In contrast, no significant association was observed between diastolic BP values and WMH burden in their diabetic counterparts (*p* > 0.05). No significant interaction effects were observed with systolic BP or MABP (interaction *p*s > 0.05).

Separate hierarchical multiple linear regression analyses examined the relationship between diabetes status and each vascular hemodynamic measure (i.e., systolic BP, diastolic BP, MABP, and PPi) on total WMH volume in both CU (Table 2) and MCI (Table 3) older adults. Covariates included in all models included age, sex, BMI, amyloid beta positivity, APOE ε4 status, and intracranial volume. Within CU older adults, the relationship vascular hemodynamic measures and WMH burden did not vary depending on diabetes status (all interaction *p*s > 0.05). In contrast, within the MCI group, diabetes status significantly interacted with PPi (β = 3.48, 95% CI: 1.52, 5.44; *p* = 0.001, partial *r* = 0.226) and diastolic blood pressure (β = –0.025, 95% CI: –0.04, –0.009; *p* = 0.002, semi-partial *r* = –0.202) on total WMH volume (Figure 1). Specifically, PPi significantly modified the relationship between diabetes status and total WMH burden (interaction *p* = 0.001), such that PPi values were associated with greater WMH burden in older adults with MCI and diabetes (β = 1.99, S.E. = 0.943, 95% CI: 0.051-3.928; *p* = 0.045, semi-partial *r* = 0.382), but not their non-diabetes counterparts (*p* > 0.05). In comparison, higher diastolic BP values were associated with greater WMH volumes in non-diabetic older adults with MCI (β = 0.008, S.E. = 0.003, 95% CI: 0.155, 2.438; *p* = 0.016, semi-partial *r* = 0.382), but not their diabetic counterparts (*p* > 0.05). There were no other significant interaction effects observed between other vascular hemodynamic measures (i.e., systolic BP, MABP) and diabetes status on total WMH burden in older adults with MCI.

**TABLE 2:** Final Multiple Linear Regression Models for CU Older Adults.

Systolic Blood Pressure						
	Estimate	SE	t-value	p-value	95%CI	Semi-partial r
Intercept	−3.827	0.521	−7.343	** < 0.001**	−4.853, −2.802	–
DM	0.373	0.686	0.544	0.587	−0.977, 1.723	0.031
SYS BP	0.004	0.002	2.520	**0.012**	0.001, 0.007	0.141
SYS BP x DM	−0.002	0.005	−0.328	0.743	−0.012, 0.008	−0.019
Age	0.025	0.004	6.499	** < 0.001**	0.017, 0.032	0.345
Gender	0.158	0.063	2.528	**0.012**	0.035, 0.281	0.142
BMI	0.007	0.005	1.312	0.191	−0.003, 0.017	0.074
CSF Aβ +	0.174	0.055	3.145	**0.002**	0.065, 0.282	0.175
APOE ε4 +	−0.019	0.055	−0.343	0.732	−0.127, 0.089	−0.019
ICV	0.001	0.000	4.813	** < 0.001**	0.001, 0.002	0.263

**Diastolic Blood Pressure**						
	**Estimate**	**SE**	**t-value**	**p-value**	**95%CI**	**Semi-partial r**

Intercept	−3.950	0.548	−7.202	** < 0.001**	−5.029, −2.870	–
DM	0.414	0.668	0.619	0.536	−0.901, 1.729	0.035
DIA BP	0.006	0.003	2.249	**0.025**	0.001, 0.012	0.126
DIA BP x DM	−0.004	0.009	−0.398	0.691	−0.021, 0.014	−0.023
Age	0.028	0.004	7.182	** < 0.001**	0.020, 0.035	0.377
Gender	0.173	0.063	2.748	**0.006**	0.049, 0.296	0.154
BMI	0.006	0.005	1.187	0.236	−0.004, 0.016	0.067
CSF Aβ +	0.158	0.056	2.826	**0.005**	0.048, 0.268	0.158
APOE ε4 +	−0.007	0.055	−0.132	0.895	−0.116, 0.101	−0.007
ICV	0.001	0.000	4.723	** < 0.001**	0.001, 0.002	0.258

**Mean Arterial Blood Pressure**						
	**Estimate**	**SE**	**t-value**	**p-value**	**95%CI**	**Semi-partial r**

Intercept	−4.067	0.546	−7.444	** < 0.001**	−5.142, −2.992	–
DM	0.493	0.820	0.601	0.548	−1.120, 2.105	0.034
MABP	0.007	0.003	2.781	**0.006**	0.002, 0.012	0.156
MABP x DM	−0.004	0.009	−0.418	0.676	−0.021, 0.013	−0.024
Age	0.026	0.004	7.030	** < 0.001**	0.019, 0.034	0.370
Gender	0.169	0.062	2.698	**0.007**	0.046, 0.291	0.151
BMI	0.006	0.005	1.134	0.258	−0.004, 0.016	0.064
CSF Aβ +	0.159	0.056	2.853	**0.005**	0.049, 0.268	0.159
APOE ε4 +	−0.013	0.055	−0.244	0.808	−0.121, 0.095	−0.014
ICV	0.001	0.000	4.764	** < 0.001**	0.001, 0.002	0.260

**Pulse Pressure Index**						
	**Estimate**	**SE**	**t-value**	**p-value**	**95%CI**	**Semi-partial r**

Intercept	−3.464	0.509	−6.801	** < 0.001**	−4.466, −2.462	–
DM	0.139	0.482	0.289	0.773	−0.810, 1.089	0.016
PPi	0.155	0.378	0.409	0.683	−0.590, 0.899	0.023
PPi x DM	0.011	1.080	0.010	0.992	−2.113, 2.135	0.001
Age	0.025	0.004	6.380	** < 0.001**	0.018, 0.033	0.340
Gender	0.158	0.063	2.489	**0.013**	0.033, 0.283	0.140
BMI	0.008	0.005	1.556	0.121	−0.002, 0.018	0.088
CSF Aβ +	0.186	0.056	3.331	**0.001**	0.076, 0.295	0.185
APOE ε4 +	−0.012	0.056	−0.212	0.832	−0.121, 0.098	-0.012
ICV	0.001	0.000	4.775	** < 0.001**	0.001, 0.002	0.261

*Abbreviations: DM, diabetes; SYS, systolic; DIA, diastolic; BP, blood pressure; MABP, mean arterial blood pressure; PPi, pulse pressure index; BMI, body mass index; CSF Aβ +, cerebrospinal fluid amyloid beta positivity; APOE ε4 +, apolipoprotein E ε4 positivity; ICV, intracranial volume. Bold values indicate for *p* ≥ 0.05.*

**TABLE 3:** Final Multiple Linear Regression Models for MCI Older Adults.

Systolic Blood Pressure						
	Estimate	SE	t-value	p-value	95%CI	Semi-partial r
Intercept	−2.420	0.584	−4.142	** < 0.001**	−3.571, −1.269	–
DM	−0.239	0.609	−0.392	0.696	−1.438, 0.961	−0.026
SYS BP	0.000	0.002	0.149	0.882	−0.004, 0.004	0.010
SYS BP x DM	0.003	0.004	0.576	0.565	−0.006, 0.011	0.038
Age	0.030	0.005	6.577	** < 0.001**	0.021, 0.039	0.400
Gender	0.061	0.074	0.825	0.410	−0.085, 0.208	0.055
BMI	−0.010	0.006	−1.690	0.092	−0.021, 0.002	−0.111
CSF Aβ +	0.122	0.069	1.773	0.078	−0.014, 0.257	0.117
APOE ε4 +	−0.055	0.066	−0.829	0.408	−0.185, 0.076	−0.055
ICV	0.001	0.000	2.839	**0.005**	0.000, 0.001	0.185

**Diastolic Blood Pressure**						
	**Estimate**	**SE**	**t-value**	**p-value**	**95%CI**	**Semi-partial r**

Intercept	−2.894	0.585	−4.950	** < 0.001**	−4.046, −1.742	–
DM	1.975	0.605	3.264	**0.001**	0.783, 3.167	0.212
DIA BP	0.007	0.003	2.243	**0.026**	0.001, 0.013	0.147
DIA BP x DM	−0.025	0.008	−3.105	**0.002**	−0.04, −0.009	−0.202
Age	0.030	0.004	6.977	** < 0.001**	0.022, 0.039	0.420
Gender	0.060	0.074	0.809	0.419	−0.086, 0.205	0.054
BMI	−0.010	0.006	−1.695	0.092	−0.021, 0.002	−0.112
CSF Aβ +	0.090	0.068	1.333	0.184	−0.043, 0.223	0.088
APOE ε4 +	−0.051	0.065	−0.797	0.426	−0.179, 0.076	-0.053
ICV	0.001	0.000	2.809	**0.005**	0.000, 0.001	0.183

**Mean Arterial Blood Pressure**						
	**Estimate**	**SE**	**t-value**	**p-value**	**95%CI**	**Semi-partial r**

Intercept	−2.792	0.603	−4.632	** < 0.001**	−3.980, −1.604	–
DM	1.232	0.720	1.712	0.088	−0.186, 2.650	0.113
MABP	0.005	0.003	1.509	0.133	−0.001, 0.011	0.100
MABP x DM	−0.012	0.008	−1.567	0.119	−0.027, 0.003	−0.103
Age	0.029	0.004	6.629	** < 0.001**	0.021, 0.038	0.403
Gender	0.063	0.075	0.843	0.400	−0.084, 0.210	0.056
BMI	−0.009	0.006	−1.608	0.109	−0.021, 0.002	−0.106
CSF Aβ +	0.107	0.068	1.564	0.119	−0.028, 0.242	0.103
APOE ε4 +	−0.049	0.066	−0.745	0.457	−0.178, 0.080	−0.049
ICV	0.001	0.000	2.851	**0.005**	0.000, 0.001	0.186

**Pulse Pressure Index**						
	**Estimate**	**SE**	**t-value**	**p-value**	**95%CI**	**Semi-partial r**

Intercept	−2.042	0.549	−3.722	** < 0.001**	−3.123, −0.961	–
DM	−1.398	0.439	−3.184	**0.002**	−2.263, −0.533	−0.207
PPi	−0.952	0.447	−2.130	**0.034**	−1.832, −0.071	−0.140
PPi x DM	3.480	0.994	3.501	**0.001**	1.521, 5.438	0.226
Age	0.033	0.004	7.301	** < 0.001**	0.024, 0.041	0.436
Gender	0.059	0.073	0.816	0.415	−0.084, 0.203	0.054
BMI	−0.011	0.006	−1.962	0.051	−0.022, 0.000	−0.129
CSF Aβ +	0.098	0.067	1.457	0.146	−0.034, 0.230	0.096
APOE ε4 +	−0.064	0.064	−0.989	0.324	−0.191, 0.063	−0.065
ICV	0.001	0.000	2.769	**0.006**	0.000, 0.001	0.181

*Abbreviations: DM, diabetes; SYS, systolic; DIA, diastolic; BP, blood pressure; MABP, mean arterial blood pressure; PPi, pulse pressure index; BMI, body mass index; CSF Aβ +, cerebrospinal fluid amyloid beta positivity; APOE ε4 +, apolipoprotein E ε4 positivity; ICV, intracranial volume. Bold values indicate for *p* ≥ 0.05.*

## Discussion

We examined the interactive effects of different vascular hemodynamic measures and diabetes on the white matter hyperintensity burden in a sample of well-characterized older adults with and without MCI. Pulse pressure index, a proxy measure for arterial stiffening and vascular non-compliance, significantly interacted with diabetes status in its association white matter hyperintensity burden, such that cognitively impaired older adults with diabetes and higher PPi values had more extensive white matter hyperintensity pathology compared to those with one or no vascular risk factors. Interestingly, these effects were only observed in older adults with MCI, and not their cognitively unimpaired counterparts. Taken together, these findings suggest that arterial stiffening and reduced vascular compliance may have a role in the presence of cerebrovascular pathology in older adults with diabetes who have objective evidence of cognitive impairment, highlighting the need for further research parsing apart the nature of the association between arterial stiffening, white matter hyperintensity burden, and diabetes status on cognitive decline in this potentially high-risk aging subgroup.

The *a priori* focus of this study was PPi given prior findings using both pulse pressure and PPi as a measure of arterial stiffness and vascular non-compliance (Peng-Lin and Yue-Chun, 2009; Nation et al., 2013, 2015, 2016), which have importance to pathological vascular aging within the context of both diabetes and cognitive impairment in late life. We also repeated all primary analyses using other vascular hemodynamic measures (i.e., systolic, diastolic, and mean arterial BP) to determine whether findings were specific to PPi. The pattern of findings observed with PPi were not observed for any other vascular hemodynamic measures, suggesting that increased arterial stiffness, but not other blood pressure measures of vascular risk, may have a unique link to the presence of greater cerebrovascular pathological burden in cognitively symptomatic older adults with diabetes. While diastolic BP was observed to significantly modify the relationship between diabetes and WMH burden, this interaction effect was such that diastolic BP was positively associated with WMH burden in non-diabetic older adults with MCI, but negatively associated (although this was statistically non-significant) with WMH burden in their diabetic counterparts. A possible explanation for this unexpected finding is that the etiology of high vs. low diastolic BP values in older adults with and without diabetes differs. For example, while high diastolic BP likely reflects general hypertension in older adults without diabetes, low diastolic blood pressure values could be more indicative of cardiac dysfunction and associated with the greater prevalence of isolated systolic hypertension due to arterial stiffening in older adults with diabetes (Franklin et al., 2011). This would represent an intriguing area for further exploration in future studies that are well-equipped to explore associations with diastolic BP and other health and brain aging variables in older adults with and without diabetes. Findings from such work would help inform how diastolic BP contributes to neurocognitive aging in an often medically complex clinical population (i.e., older adults with diabetes and cognitive impairment).

There are a variety of vascular risk factors (e.g., hypertension, atherosclerosis, hyperlipidemia, metabolic syndrome, diabetes) that have been linked to pathological brain aging processes, such as evidence of cerebrovascular disease on neuroimaging (e.g., white matter hyperintensity burden, altered cerebral perfusion), neuropathological changes on brain autopsy, cognitive impairment, and functional decline (Arvanitakis et al., 2004; de la Torre, 2010; Crane et al., 2013; Bangen et al., 2018; Werhane et al., 2018; Thomas et al., 2020). There is a growing body of evidence to suggest a robust association between arterial stiffness and pathological changes associated with advanced age, including gray and white matter degradations, accumulation of AD neuropathology, and cognitive decline (Nation et al., 2015; Saji et al., 2016). It is thought that arterial stiffness gives rise to these changes by inducing a deleterious systemic hypertensive state, causing downstream damage to vulnerable microvasculature and associated tissue. The brain is especially susceptible to these effects given the high density and low impedance of its microvasculature, which allows for the increased pulsatile load that occurs in the context of arterial stiffening to deeply penetrate the microvascular bed and cause damage to the vessels (particularly in watershed areas with low perfusion), ultimately leading to chronic hypoperfusion and ischemia that harms the surrounding brain tissue (Watson et al., 2011; Wardlaw et al., 2013; Nation et al., 2015). Evidence in support of this mechanism comes from studies linking arterial stiffening to morphological and functional brain change as early as young and mid-life (Tsao et al., 2013; Maillard et al., 2016; Pase et al., 2016), highlighting the chronic, insidious contribution of vascular disease to cognitive aging. Our findings extend this literature by demonstrating that elevated arterial stiffness, as measured by pulse pressure index values, is particularly deleterious in the presence of diabetes and MCI.

While the role of white matter alterations in cognitive aging has long been acknowledged, there is a growing body of evidence highlighting their role in the link between diabetes and late-life cognitive impairment. White matter hyperintensities are more prevalent in populations with increased vascular risk, such as those with diabetes, and several studies have shown that white matter hyperintensity burden predicts cognitive impairment and MCI in older adults (DeCarli et al., 1995; Au et al., 2006; Delano-Wood et al., 2008, 2009; Silbert et al., 2008, 2009; Bangen et al., 2018), more rapid cognitive decline within at-risk populations (Tosto et al., 2014), and the onset and progression of incident AD dementia (Wolf et al., 2000; Brickman et al., 2012; Brickman et al., 2015). Critically, findings from the present study show that, for individuals already at increased risk for developing dementia (i.e., those with MCI), diabetes in the presence of increased systemic vascular pathology is associated with greater white matter hyperintensity burden. Such findings not only suggest that arterial stiffening may contribute to the development of cerebrovascular pathology in at-risk older adults, but moreover underscore its possible utility as an early, potentially modifiable risk factor for cognitive impairment. Given the specificity of these findings to MCI, future exploration of the sensitivity and utility of sensitive brain markers of vascular insufficiency that may precede gross, end stage white matter changes observable on MRI is also warranted, as such markers may allow for the detection of similar interactive effects of arterial stiffness and diabetes within cognitively unimpaired individuals. Such research may lead to a better understanding of the pathophysiological mechanism that underlies the relationship between arterial stiffening and white matter pathogenesis in the context of diabetes, and possible targets for intervention prior to the development of cognitive impairment.

There are several strengths to the present study. This well-characterized older adult sample allowed us to adjust for multiple relevant covariates to increase confidence in the interpretation of the effects of arterial stiffness and diabetes. To this point, we have confidence in the robustness of the reported findings given that they held across various models adjusted for several relevant factors and confounds. We also identified cognitively unimpaired older adults and those with MCI within the ADNI dataset using highly sensitive diagnostic criteria that have shown to be more reliable and stable than the conventional diagnostic criteria for MCI and yield far fewer (∼33%) false positives diagnostic errors (Jak et al., 2009; Bondi et al., 2014; Edmonds et al., 2015, 2016). However, there are some important limitations to the present study that must also be considered. The ADNI exclusion and inclusion criteria yielded a particularly healthy sample (on average, participants were within recommended ranges clinically for all vascular hemodynamic measures) that likely does not adequately mirror the health status of the general population. Indeed, only 9.5% of our sample met criteria for diabetes which, while comparable to diabetes prevalence in the American population, resulted in a relatively small cell size in our final diabetes analytic sample. This sample may therefore reflect a subsample of older adults with and without diabetes that has relatively reduced chronic disease and vascular risk burden compared to the general population. This more restricted range of vascular risk may also explain why PPi was not associated with WMH in our sample overall. Additionally, our sample was highly educated (average was approximately 16 years of education, while the national average for older adults age 65 + is 13 years [Ryan and Bauman, 2016]) and homogeneous with respect to race/ethnicity (predominantly White). This is an important caveat this study, particularly given ample literature documenting differences across racial/ethnics groups in diabetes prevalence and outcomes (Walker et al., 2016). Thus, our findings may apply only to a demographically specific subpopulation (i.e., highly educated older adults who identify as White and are willing and have access to participate in an Alzheimer’s disease study) with a relatively restricted range of vascular disease. Additional research using more diverse, multiethnic epidemiological samples would therefore facilitate an enhanced understanding of how relationships between these variables compare across subpopulations, and moreover clarify important mechanisms that may underlie observed differences across demographic and clinical groups. Additionally, there was limited information available for this sample regarding other risk and protective factors for arterial stiffness and vascular disease (e.g., hemoglobin A_1__*C*_ levels, disease characteristics, disease management, health behaviors). Thus, replication of these results in a more representative community sample that is well characterized with respect to vascular risk is needed in order to further elucidate the role of arterial stiffness as a predictor of white matter pathology in aging individuals with DM. Finally, our study design was cross-sectional in nature, and thus did not address questions about directionality of these relationships nor how they might evolve over time. Future research that employs multivariate models to explore more complex associations between longitudinal pulse pressure index measurements, diabetes status, and white matter pathology is needed.

## Conclusion

Our results demonstrated that higher pulse pressure index values in the presence of diabetes was associated with small vessel disease as indexed by white matter hyperintensities in older adults with MCI. These findings have important implications for the early identification of individuals at risk for progressive cognitive decline and dementia, as well as the development of treatment targets to potentially stave off cognitive decline in order to optimize independence and quality of life in at-risk older adults. The proportion of people over the age of 65 is rapidly increasing in our population and the number of individuals with dementia are expected to burgeon in tandem with this population expansion. Such demographic changes will stretch resources related to the social, economic, and psychological needs of those who develop cognitive impairment in late life. While we do not yet have therapies that can reliably slow or halt cognitive decline, there are a variety of validated preventative approaches and interventions that can target vascular risk factors. Thus, identifying high-risk individuals using potentially modifiable markers, such as elevated pulse pressure index values (which is a relatively quick, cost-effective, and non-invasive proximate measure of arterial stiffness), prior to the onset of cognitive impairment may help to relieve this burden and promote independence in late adulthood, particularly in populations where vascular risk is elevated. Future longitudinal studies that include biomarkers are needed in order to clarify the etiology and timeline of the association between pulse pressure index values and different brain aging processes. Moreover, research that extends into middle age (and focuses on earlier disease states such as prediabetes) will assist in further exploring the utility of the pulse pressure index as an early marker of risk for poor cognitive outcomes in aging.

## Data Availability Statement

The data analyzed in this study is subject to the following licenses/restrictions: The datasets analyzed for this study can be found in the Alzheimer’s Disease Neuroimaging Initiative (ADNI) database. Investigators must submit an application to access ADNI data. Requests to access these datasets should be directed to the official ADNI website: adni.loni.usc.edu.

## Ethics Statement

The studies involving human participants were reviewed and approved by the ethics committees/institutional review boards that approved the ADNI, including: Albany Medical Center Committee on Research Involving Human Subjects Institutional Review Board, Boston University Medical Campus and Boston Medical Center Institutional Review Board, Butler Hospital Institutional Review Board, Cleveland Clinic Institutional Review Board, Columbia University Medical Center Institutional Review Board, Duke University Health System Institutional Review Board, Emory Institutional Review Board, Georgetown University Institutional Review Board, Health Sciences Institutional Review Board, Houston Methodist Institutional Review Board, Howard University Office of Regulatory Research Compliance, Icahn School of Medicine at Mount Sinai Program for the Protection of Human Subjects, Indiana University Institutional Review Board, Institutional Review Board of Baylor College of Medicine, Jewish General Hospital Research Ethics Board, Johns Hopkins Medicine Institutional Review Board, Lifespan - Rhode Island Hospital Institutional Review Board, Mayo Clinic Institutional Review Board, Mount Sinai Medical Center Institutional Review Board, Nathan Kline Institute for Psychiatric Research and Rockland Psychiatric Center Institutional Review Board, New York University Langone Medical Center School of Medicine Institutional Review Board, Northwestern University Institutional Review Board, Oregon Health and Science University Institutional Review Board, Partners Human Research Committee Research Ethics, Board Sunnybrook Health Sciences Centre, Roper St. Francis Healthcare Institutional Review Board, Rush University Medical Center Institutional Review Board, St. Joseph’s Phoenix Institutional Review Board, Stanford Institutional Review Board, The Ohio State University Institutional Review Board, University Hospitals Cleveland Medical Center Institutional Review Board, University of Alabama Office of the IRB, University of British Columbia Research Ethics Board, University of California Davis Institutional Review Board Administration, University of California Los Angeles Office of the Human Research Protection Program, University of California San Diego Human Research Protections Program, University of California San Francisco Human Research Protection Program, University of Iowa Institutional Review Board, University of Kansas Medical Center Human Subjects Committee, University of Kentucky Medical Institutional Review Board, University of Michigan Medical School Institutional Review Board, University of Pennsylvania Institutional Review Board, University of Pittsburgh Institutional Review Board, University of Rochester Research Subjects Review Board, University of South Florida Institutional Review Board, University of Southern, California Institutional Review Board, UT Southwestern Institution Review Board, VA Long Beach Healthcare System Institutional Review Board, Vanderbilt University Medical Center Institutional Review Board, Wake Forest School of Medicine Institutional Review Board, Washington University School of Medicine Institutional Review Board, Western Institutional Review Board, Western University Health Sciences Research Ethics Board, and Yale University Institutional Review Board. The patients/participants provided their written informed consent to participate in this study.

## Author Contributions

MW, KB, KT, ES, and LD-W contributed to conception and design of the study. KT and AW organized the database. MW performed the statistical analysis and wrote the manuscript. MB and EE provided consultation regarding MCI diagnosis using Jak et al. diagnostic criteria to identify CU and MCI participants in the ADNI database. DN provided consultation and guidance with respect to the use of PP as a measure for arterial stiffness and its application to the ADNI data. All authors contributed to manuscript revision, read, and approved the submitted version.

## Conflict of Interest

The authors declare that the research was conducted in the absence of any commercial or financial relationships that could be construed as a potential conflict of interest.

## Publisher’s Note

All claims expressed in this article are solely those of the authors and do not necessarily represent those of their affiliated organizations, or those of the publisher, the editors and the reviewers. Any product that may be evaluated in this article, or claim that may be made by its manufacturer, is not guaranteed or endorsed by the publisher.

## Footnotes

1
http://adni.loni.usc.edu/methods/mri-tool/mri-acquisition/

2 Clinical cut points: hypertension, BP > 140/90 in older adults < 60 years of age and 150/90 in older adults of 60 + years of age (James et al., 2014); functional dependence, FAQ ≥ 6 (Teng et al., 2010); presence of depressive symptoms, GDS > 9 (Yesavage and Sheikh, 1986); cognitive impairment, MMSE < 24 (Folstein et al., 1975).
